# Optimizing Management of Morton’s Neuroma: Extracorporeal Shock Wave Therapy in Clinical Practice

**DOI:** 10.7759/cureus.101168

**Published:** 2026-01-09

**Authors:** Kensuke Nakamura

**Affiliations:** 1 Department of Rehabilitation, Podiatric Surgery &amp; Medicine Clinic Ogikubo, Suginami, JPN

**Keywords:** extracorporeal shock wave therapy, focused extracorporeal shock wave therapy, morton’s neuroma, narrative reviews, neuropathy

## Abstract

Morton’s neuroma (MN) is frequently refractory to conventional conservative therapies, and surgical excision carries the risk of recurrent pain. While extracorporeal shock wave therapy (ESWT) has emerged as a potential non-invasive intervention for MN, a standardized clinical protocol for its application remains to be established. This review aims to elucidate the physiological validity of ESWT for MN and propose an optimized clinical protocol. Existing randomized controlled trials of ESWT for MN were analyzed, and findings from other entrapment neuropathies were extrapolated to critically appraise interventional factors influencing therapeutic outcomes. ESWT exerts a biphasic effect: immediate analgesia via the degeneration of free nerve endings, and radical pathophysiological improvement through the promotion of neovascularization and tissue repair. The present analysis suggests that the use of local anesthesia may inhibit mechanotransduction, potentially reducing success rates. Furthermore, considering the dynamics of growth factors involved in tissue regeneration, multiple sessions at one-week intervals are more rational than a single application. Optimizing ESWT through repetitive sessions without anesthesia at the maximum tolerable energy flux density may maximize therapeutic efficacy. ESWT is expected to become an integral, minimally invasive standard therapy that precludes the necessity for surgical intervention.

## Introduction and background

Morton’s neuroma (MN) is a common compressive plantar neuropathy characterized primarily by interdigital pain [1]. Although termed a "neuroma," MN is not a neoplastic lesion but a non-neoplastic benign condition involving perineural fibrotic thickening [1]. Clinically, the highest incidence occurs in the third web space, followed by the second [2]. Epidemiologically, the condition predominantly affects adults in their 40s to 60s, with a higher prevalence in female patients than in males [2]. According to Latinovic et al., the incidence per 100,000 person-years is 50.2% for males and 87.5% for females, ranking it as one of the most frequent compressive neuropathies after carpal tunnel syndrome [3]. The clinical manifestations of MN include forefoot symptoms such as sharp pain, burning sensations, and paresthesia in the affected interdigital space [4]. While the exact pathogenesis of MN remains to be fully elucidated, the most widely accepted theory suggests that the nerve passing beneath the deep transverse metatarsal ligament is subjected to mechanical compressive stress due to specific anatomical characteristics of the foot. This leads to the accumulation of chronic micro-injuries, which subsequently induce degeneration of the perineural tissues [1,5,6]. Furthermore, ischemic changes in the nerve and the involvement of intermetatarsal bursitis have been implicated, suggesting a multifactorial etiology [5,7].

The diagnosis is primarily based on clinical findings, with the reproduction of symptoms through palpation being a critical component. In particular, Mulder’s test, which involves the elicitation of a palpable click and the reproduction of pain upon lateral compression of the metatarsal heads, serves as a vital clinical indicator that corroborates the diagnosis [4,5]. Regarding diagnostic imaging, ultrasonography is highly effective and non-invasive, with reported sensitivities reaching approximately 95% [8]. Furthermore, the concomitant use of magnetic resonance imaging facilitates the identification of deep-seated lesions that may be challenging to evaluate via ultrasound, and enables a more precise differential diagnosis from other forefoot disorders [8].

The primary therapeutic approach for MN involves conservative management, including footwear education, use of metatarsal pads, and activity modification [5]. In cases refractory to these initial interventions, local injections of corticosteroids or anesthetics are widely administered. A recent study by Lee et al. reported that ultrasound-guided corticosteroid injections provide favorable short-term pain relief [1]. However, their long-term efficacy remains questionable; a randomized controlled trial by Mahadevan et al. indicated that the treatment failure rate reached approximately 50% within one year post-injection [9]. Furthermore, the administration of frequent corticosteroid injections necessitates cautious clinical judgment due to the risk of irreversible adverse effects, such as atrophy of the plantar fat pad, degeneration of the digital nerves, instability of the metatarsophalangeal joints, and cutaneous hypopigmentation [9].

Surgical excision of the neuroma is considered the final therapeutic option for cases refractory to conservative management. To bridge the gap between conservative care and surgery, several interventional therapies have been explored, including botulinum toxin A injections [10], radiofrequency ablation [11], ultrasound-guided cryoablation [12], and platelet-rich plasma (PRP) [13]. However, these interventions still involve varying degrees of invasiveness, and a lack of robust comparative studies with conventional conservative management remains a challenge. While surgery is generally considered an effective intervention, significant long-term challenges remain. A follow-up study by Kasparek & Schneider, with an average duration of 15 years, reported that although overall postoperative satisfaction was favorable, approximately 8% of patients experienced recurrent pain originating from stump neuromas [14]. Furthermore, a prospective study by Bucknall et al. revealed that complete pain relief was achieved in only 63% of cases, with approximately 25% of patients failing to show improvements in activities of daily living [15]. Given the risks associated with surgical invasion and functional limitations, there is an urgent clinical need to establish minimally invasive alternative therapies that can preclude surgery.

In recent years, extracorporeal shock wave therapy (ESWT) has garnered significant attention as a non-invasive treatment for musculoskeletal disorders. While ESWT has a robust evidence base for enthesopathies such as plantar fasciitis, its application in entrapment neuropathies, including MN, has only recently been reported [16,17]. The suggested physiological mechanisms of ESWT include pain reduction through the degeneration of free nerve endings (nociceptors) and the promotion of neovascularization and tissue repair [18,19]. Additionally, systematic reviews of MN have identified ESWT as a promising therapeutic option [20]. However, clinical research remains limited to two primary studies conducted by Seok et al. and Fridman et al. [16,21]. Consequently, standardized treatment protocols and indications have yet to be established. The purpose of this review is to integrate these limited clinical data and provide an original re-interpretation (incorporating insights from other entrapment neuropathies) to present the physiological validity of ESWT for MN, and propose guidelines for its optimal clinical implementation.

## Review

Methodology

A comprehensive literature search was conducted across multiple electronic databases, including PubMed and Google Scholar, to identify relevant studies published up to December 1, 2025. The search strategy utilized combinations of keywords such as "Morton’s neuroma," "Extracorporeal Shock Wave Therapy," "ESWT," "Focused extracorporeal shock wave therapy," and "Radial extracorporeal shock wave therapy." Priority was given to clinical studies investigating the use of ESWT for MN, specifically focusing on peer-reviewed original articles. Exclusion criteria included studies unrelated to the scope of this review, non-peer-reviewed articles, and literature published in languages other than English. 

The selection of studies was conducted through a multi-stage screening process, beginning with a review of titles and abstracts, followed by a full-text evaluation to ensure adherence to the predefined eligibility criteria. In an effort to minimise bias, each identified study was subjected to a critical appraisal of its methodological quality, with particular attention paid to study design, participant characteristics, and the clarity of treatment protocols. Based on the search results, two primary studies (randomized controlled trials) were identified as eligible, and their findings were integrated into this narrative review to synthesize the existing evidence. Due to the limited number of identified trials and the heterogeneity in their treatment protocols and outcome measures, a quantitative meta-analysis was not performed; instead, a qualitative synthesis of the evidence was conducted.

Integration of evidence and critical appraisal

Analysis of Primary Literature

To date, randomized controlled trials (RCTs) verifying the efficacy of ESWT for MN remain limited to two studies: those by Seok et al. and Fridman et al. (Table 1) [16,21].

**Table 1:** Summary of randomized controlled trials on ESWT for Morton’s neuroma ESWT: extracorporeal shock wave therapy, AOFAS: American Orthopedic Foot and Ankle Society, VAS: Visual Analogue Scale

Document	Subjects	Energy setting	Number of shocks	Number of sessions	Local anesthesia	Evaluation period	Pain	Functional assessment
Fridman et al. (2009) [21]	25 feet (13 intervention)	21 kV	2,000	Single	Yes	12 weeks	Significant VAS improvement	Not performed
Seok et al. (2016) [16]	26 patients (14 intervention)	0.12–0.24 mJ/mm²	1,000	Single	No	4 weeks	Significant VAS improvement	Significant AOFAS score improvement

Fridman et al. conducted a double-blind RCT involving 25 feet, administering a single session of 2,000 shocks at an energy setting of 21 kV to the affected area [21]. Their findings demonstrated a significant improvement in visual analogue scale (VAS) scores at 12 weeks post-treatment [21]. However, it should be noted that complete pain resolution was achieved in only four out of the 13 patients (approximately 30%) in the intervention group. This underscores the necessity for a cautious evaluation of the efficacy limits before positioning ESWT as a definitive alternative to surgical excision.

Conversely, Seok et al. performed a study on 14 participants using a single session of 1,000 shocks at the maximum tolerable energy level (0.12-0.24 mJ/mm2) [16]. At four weeks post-irradiation, they reported not only a significant reduction in VAS scores, but also improvements in the American Orthopedic Foot and Ankle Society (AOFAS) lesser toes scale, a validated measure of foot function [16].

These pioneering studies suggest that the benefits of ESWT extend beyond subjective pain relief, contributing to functional improvements in gait capacity and overall quality of life. Based on the findings of these two reports, ESWT is emerging as a novel, non-invasive, and conservative option for refractory MN. Nevertheless, further critical examination regarding optimal irradiation parameters and long-term prognosis, which is discussed in the following section, remains imperative.

Critical Appraisal of Interventional Factors Influencing Therapeutic Efficacy

A detailed comparison of these two studies revealed three primary clinical challenges that are essential for optimizing ESWT for MN.

The first is the physiological impact of local anesthesia. Fridman et al. administered local anesthesia to mitigate procedural pain, whereas Seok et al. performed treatment without anesthesia [16,21]. Klonschinski et al. reported that the use of local anesthesia significantly altered the physiological response to ESWT [22]. Similarly, Rompe et al. suggested that irradiation under local anesthesia may reduce the overall success rates [23]. This indicates that local anesthetics may inhibit mechano-transduction at the cellular level, thereby attenuating the intrinsic tissue repair effects of ESWT. Therefore, to maximize the therapeutic outcomes, the procedure should ideally be performed without anesthesia.

The second challenge is the impact of the total number of shocks. Fridman et al. applied 2,000 shocks, whereas Seok et al. applied 1,000 [16,21]. Although direct comparison is challenging due to differing evaluation scales and follow-up periods, the reduction in pain scores was 4.7 points (numerical rating scale: NRS) in the Fridman et al. study and 28.3 mm (VAS) in the Seok et al. study [16,21]. In a study on knee osteoarthritis, Zhang et al. pointed out that clinical outcomes may depend more on the energy flux density than on the total number of shocks [24]; conversely, Yang et al. reported that an increased number of shocks contributes to the improvement of soft tissue properties [25]. Given that the fundamental pathology of MN involves perineural fibrotic thickening, ensuring an adequate number of shocks within the patient's tolerance level may be clinically beneficial for providing sufficient energy to thickened tissues.

The third challenge is the limitation of session frequency. Both studies reported outcomes following a single irradiation session, leaving the efficacy of multiple sessions unelucidated for MN [16,21]. However, evidence from other conditions suggests a benefit from repeated sessions. Aldajah et al. reported significant improvements after five or more sessions [26], and Gür et al. demonstrated the superiority of multiple sessions for myofascial pain syndrome [27]. Considering the chronic fibrosis and degenerative processes inherent in MN, continuous stimulation through multiple sessions is likely to promote tissue repair and suppress the regeneration of free nerve endings, thereby contributing to the maintenance of long-term analgesic effects [28].

Risk-Benefit Comparison With Conventional Conservative Therapies

Among the standard conservative treatments for MN, corticosteroid injections in the affected interdigital space are widely utilized. Although corticosteroid injections provide immediate short-term pain relief, their sustained efficacy is significantly limited, with treatment failure rates reaching 50% within one year [9]. Furthermore, repetitive administration poses the risk of irreversible adverse effects that can severely impair activities of daily living, including atrophy of the plantar fat pad (which serves as a crucial cushion during weight-bearing) and toe deformities resulting from instability of the metatarsophalangeal (MTP) joints [9].

In contrast, the hallmark of ESWT is its ability to induce micro-trauma in fibrotic or degenerated tissues, thereby triggering a regenerative tissue repair [19,29]. Regarding safety, ESWT has been demonstrated to be an extremely low-risk intervention. According to prior studies, the majority of side effects are limited to transient skin erythema or mild procedural pain, with almost no reports of serious or long-term complications [30,31]. Although Haake et al. reported rare systemic reactions such as migraines or syncope following irradiation, these instances were transient and followed by rapid recovery [31].

Taken together, by circumventing the risk of irreversible tissue damage associated with corticosteroid injections, while promoting the repair of underlying tissue degeneration, ESWT has high clinical validity as a potential first-line therapy for refractory MN.

Extrapolation From Evidence in Other Entrapment Neuropathies

Although clinical studies specifically addressing ESWT for MN are currently limited to two reports, extrapolating findings from other entrapment neuropathies with analogous pathologies is highly valuable for reinforcing the therapeutic rationale. Representative conditions include carpal tunnel syndrome (CTS) and cubital tunnel syndrome [32,33].

Gesslbauer et al. reported that ESWT in 30 patients with CTS resulted in significant improvements not only in VAS scores and grip strength, but also in sensory nerve conduction velocity, which is a key physiological parameter [32]. Similarly, Shen et al. observed improvements in VAS scores and the Disabilities of the Arm, Shoulder, and Hand (QuickDASH) score, which is a validated upper-extremity functional scale, in a study involving 10 elbows from seven patients with cubital tunnel syndrome [33].

Regarding the mechanisms underlying symptom relief in these entrapment neuropathies, it has been suggested that ESWT may reduce endo-neurial compartment pressure and enhance microcirculation within ischemic nerve tissues [34]. Furthermore, Park et al. demonstrated in an animal model that ESWT irradiation facilitates peripheral nerve remyelination and induces Schwann cell plasticity [35].

Given that the core pathology of MN involves physical compression by the deep transverse metatarsal ligament and subsequent perineural ischemia and fibrosis, the therapeutic mechanisms observed under these conditions are highly consistent and applicable to MN. These findings provide robust support for the clinical validity of ESWT in the management of Morton’s neuroma.

Physiological mechanisms of ESWT in Morton’s neuroma

Analgesic Mechanisms: Alterations in Nociceptors and Neurophysiological Pathways

The analgesic mechanisms of ESWT can be elucidated through anatomical alterations in nociceptors and neurophysiological modulatory effects.

Regarding direct impacts on nociceptors, Ohtori et al. histologically demonstrated that ESWT irradiation selectively destroys and induces degeneration of free nerve endings [18]. Hausdorf et al. reported a significant and selective reduction in unmyelinated nerve fibers, which are the primary conduits for chronic pain transmission following ESWT [36]. Additionally, Takahashi et al. suggested that repetitive ESWT sessions could suppress the reinnervation of these free nerve endings, potentially maintaining long-term analgesic effects [28]. This process suggests that ESWT physically disrupts sensitized neural networks, followed by the restoration of normal sensory input during reinnervation.

Secondly, neurophysiological changes involving the dynamics of pain-transmitting substances play a critical role. Maier et al. reported that ESWT irradiation triggers the transient release of Substance P, a key algogenic peptide, which ultimately leads to its depletion within the nerve terminals [37]. Furthermore, Takahashi et al. revealed that ESWT inhibited the expression of calcitonin gene-related peptide (CGRP) in the dorsal root ganglion and suppressed pain transmission mediated by prostaglandins [38].

Based on these collective findings, it is inferred that ESWT has a sustained inhibitory effect on pain, which is challenging to achieve with conventional conservative therapies. This efficacy is likely derived from the synergistic interplay between the direct deafferentation of nociceptors and the indirect modulation of pain-related neuropeptide dynamics.

Tissue Repair Mechanisms and Amelioration of Ischemic Lesions

The pathological background of MN is characterized by impaired endoneurial microcirculation resulting from fibrosis and degeneration of perineural tissues. Chronic entrapment by the deep transverse metatarsal ligament induces intimal arterial thickening and thrombus formation, subjecting the digital nerve to a state of chronic ischemia [5,7]. This ischemic environment not only accelerates the irreversible degeneration of perineural structures but also serves as a critical factor in the chronicity of pain.

Wang et al. reported that ESWT irradiation, mediated by mechano-transduction, upregulates angiogenic factors such as eNOS and PCNA, promoting microcirculatory remodeling [19]. Furthermore, Berta et al. observed that the expression of transforming growth factor-beta 1 (TGF-β1) and types I and III collagen is upregulated starting from day 6 post-irradiation [39]. These findings suggest that ESWT may improve the local microcirculation and hemodynamics, thereby facilitating the remodeling of degenerated sites. In the context of MN, this mechanism is thought to drastically enhance the supply of oxygen and nutrients to the ischemic nerve tissue, while contributing to the rapid clearance of accumulated algogenic substances.

Mitigation of Physical Compressive Stress

Although the precise etiology of MN remains unclear, it is widely postulated that chronic mechanical stress exerted by the deep transverse metatarsal ligament (DTML) is the primary factor that induces perineural thickening and fibrosis [1,5,6]. The potential of ESWT to mitigate this physical compression can be inferred from the prior findings of analogous entrapment neuropathies.

Gesslbauer et al. reported that the efficacy of ESWT in carpal tunnel syndrome may be attributed to its anti-inflammatory effects, which reduce the endoneurial compartment pressure and promote nerve regeneration [32]. Applying this insight to the anatomical configuration of the MN, it is highly probable that ESWT exerts an anti-inflammatory effect on soft tissues, such as the DTML and intermetatarsal bursa. This leads to a reduction in the internal pressure surrounding the nerve tissue, thereby contributing to the resolution of clinical symptoms.

Proposed clinical protocols for ESWT in Morton’s neuroma

Optimization of Irradiation Protocols

While Seok et al. and Fridman et al. reported significant pain reduction and functional improvement following a single session, evidence extrapolated from other musculoskeletal disorders suggests that a course of approximately five consecutive interventions is recommended (Table 2) [16,21,26,27]. To maintain the peak of tissue repair, a weekly treatment interval is recommended, aligning with the temporal peak expression of regenerative markers such as TGF-β1 (Table 2) [39].

**Table 2:** Proposed optimized ESWT protocol for Morton’s neuroma compared with existing practices ESWT: extracorporeal shock wave therapy

Item	Number of sessions	Treatment interval	Local anesthesia	Energy level	Adjunctive therapy
Existing protocols [16,21]	Single session	N/A	Used in some cases	Constant or maximum tolerable level	Not mentioned
Proposed protocol (this review)	Approximately 5 repeated sessions	1-week interval	Without anesthesia (in principle)	Maximum intensity within patient tolerance	Combination with physical therapy, etc.
Rationale/objective	Sustained tissue repair and suppression of nerve regeneration [26,28]	Align with peak expression of TGF-beta 1, etc. [39]	Avoidance of mechanotransduction inhibition [22]	Penetration depth into fibrotic tissues and promotion of repair [24]	Combination with physical therapy (e.g., stretching) and orthotic devices (e.g., metatarsal pads), leading to improved treatment success rates [40,41]

Given that the fundamental pathology of MN involves tissue fibrosis and degeneration, it is advisable to employ the maximum energy flux density (EFD) within the patient's tolerance. Zhang et al. suggested that higher energy densities were more likely to induce symptomatic improvement [24]. However, as high-intensity irradiation may rarely trigger vasovagal reactions such as headaches or syncope, continuous monitoring of the patient’s systemic status during the procedure is indispensable. Although a minimum of 1,000 shocks per session is generally regarded as the threshold for clinical improvement, the possibility that an increased number of shocks can contribute to soft tissue remodeling, as reported by Yang et al., should be considered [16,25]. Tailoring the total shock count based on individual symptomatic responses remains an important subject for future investigations.

Positioning and Technical Considerations for ESWT

Although previous studies have not addressed positioning in detail, the supine or prone position is clinically recommended to facilitate muscle relaxation and ensure stable irradiation. For precise targeting, it is essential to pre-identify the size, depth, and anatomical location of the neuroma using ultrasonography. Once the depth of the lesion is confirmed, selecting the appropriate device and adjusting the probe offset to ensure that the focal point properly converges on the neuroma are critical steps in enhancing the accuracy and efficacy of the treatment.

Considerations Regarding the Use of Local Anesthesia

ESWT should be performed without anesthesia (Table 2). This recommendation is based on evidence suggesting that local anesthetics may inhibit mechano-transduction at the cellular level, potentially limiting subsequent tissue repair. However, in patients with exceptionally low pain thresholds, the pain associated with irradiation may pose a risk of triggering adverse events. Given that Fridman et al. reported significant pain reduction even with the concomitant use of local anesthesia [21], its administration may be considered a pragmatic option for patients who cannot otherwise tolerate the procedure, thereby ensuring treatment completion.

Indications and Selection Criteria for ESWT

ESWT is recommended for MN cases that show insufficient clinical improvement following three to six months of initial conservative management, which should prioritize physical therapy alongside footwear modification and activity adjustment [16,21]. To maximize the probability of treatment success, a multimodal approach is preferable to monotherapy. As demonstrated by Burton et al. and Pham et al., combining ESWT with physical therapy or other conservative interventions is expected to yield synergistic effects on pain reduction and functional recovery [40,41]. Extrapolating from these findings, a similar synergistic benefit is expected in MN when ESWT is combined with specific protocols, including stretching of the plantar fascia and triceps surae, intrinsic foot muscle strengthening, and orthotic management (e.g., metatarsal pads) to decompress the intermetatarsal space.

Future perspectives and challenges

While ESWT represents a promising non-invasive option for reducing pain and promoting tissue repair in MN, several challenges must be addressed to establish it as a standardized treatment modality.

First, the validation of long-term outcomes is essential. Existing studies have primarily focused on short-term evaluations ranging from four to 12 weeks [16,21]. Future research must investigate long-term recurrence rates and patient satisfaction beyond one year post-irradiation, as well as the rate of successful avoidance of surgical intervention.

Second, the correlation between neuroma morphology, size, and therapeutic efficacy warrants further clarification. Previous studies have not addressed how the maximum diameter or cross-sectional area of a neuroma, measured by ultrasonography or magnetic resonance imaging, influences treatment outcomes [16,21]. Determining whether ESWT remains effective for lesions exceeding a specific size or identifying a threshold that necessitates a transition to surgical intervention is crucial for clinical decision-making.

Third, a comparative analysis based on the different physical characteristics of ESWT devices is required. Current knowledge is limited to focused ESWT; however, the efficacy of radial ESWT, which distributes stimuli over a wider and more superficial area, remains unclear. Therefore, it is necessary to compare how differences in device types affect clinical outcomes and cost-effectiveness.

Despite these challenges, ESWT possesses unique characteristics, namely its minimal invasiveness and regenerative potential, that distinguish it from conventional therapies. Through the formulation of optimal protocols through large-scale multicenter collaborative studies, ESWT is expected to become an integral component of the standard treatment algorithm for Morton’s neuroma.

## Conclusions

MN presents a significant clinical challenge; conventional conservative therapies often yield limited efficacy, yet surgical intervention does not always guarantee satisfactory outcomes. In contrast, ESWT has emerged as a highly valuable, non-invasive treatment that uniquely combines immediate analgesic effects, mediated by the degeneration of free nerve endings, with fundamental pathophysiological improvements through the promotion of neovascularization and tissue repair.

By optimizing the clinical implementation, specifically through the application of multiple sessions without anesthesia and the use of high energy flux density, as proposed in this review, the success rate of ESWT for MN can be further enhanced. ESWT is anticipated to become an integral component of standardized, minimally invasive care, potentially precluding surgical intervention and gaining widespread adoption in clinical practice.
